# Variation of resection margins in oral cancer in dependence of tumor stage and subsite – a retrospective cohort study

**DOI:** 10.1007/s00784-024-05711-5

**Published:** 2024-05-20

**Authors:** Julius Moratin, Dominik Horn, Marcel Oehme, Karl Semmelmayer, Christa Flechtenmacher, Oliver Ristow, Thomas Held, Michael Engel, Jürgen Hoffmann, Christian Freudlsperger

**Affiliations:** 1 Department of Oral and Cranio-Maxillofacial Surgery, University of Heidelberg, Im Neuenheimer Feld 400, 69120 Heidelberg, Germany; 2 Department of Oral and Maxillofacial Surgery, Saarland University Hospital, Kirrberger Straße, 66424 Homburg, Germany; 3 Institute of Pathology, University of Heidelberg, Im Neuenheimer Feld 224, 69120 Heidelberg, Germany; 4 Department of Radiation Oncology, University of Heidelberg, Im Neuenheimer Feld 400, 69120 Heidelberg, Germany

**Keywords:** HNSCC, Oral squamous cell carcinomas, Resection margins, Survival

## Abstract

**Objectives:**

Surgical resection is a key component of the treatment of head and neck cancer and the achievement of free surgical margins are essential for the patients’ outcome in terms of survival. While there is a general recommendation for a free resection range of 5 mm, up to date, there is a lack of investigations on the quality of tumor resection in dependence of affected subsite and tumor stage. In the presented study, predictors for the achieved resection margins in surgically treated oral squamous cell carcinomas were analyzed.

**Materials and methods:**

A cohort of 567 patients was included in a retrospective analysis and resection status with exact margin ranges were analysed. Tumor stage, affected subsite and the results of the intraoperative frozen section analysis were assessed. Primary endpoint was the achieved resection margin in mm, secondary endpoints were overall and progression-free survival.

**Results:**

The observed mean values of minimal resection margins differed significantly between the investigated subsites (*p* = 0.042),pathological tumor stages (*p* < 0.001) and in tumors which demonstrated perineural infiltration (Pn1, *p* = 0.002). Furthermore, there was a significant impact of the results of the intraoperative frozen section analysis on progression-free and overall survival (*p* < 0.001).

**Conclusions:**

Our data clearly indicate that resection status differs between tumors of different subsites and tumor stages.

**Clinical relevance:**

Clinical procedures should be adapted in order to achieve similar certainty in all resections, and, thus to improve patients’ outcome.

## Introduction

Squamous cell carcinomas of the oral cavity (OSCC) is characterized by clinical heterogeneity [1]. While primary surgical therapy is the first-line standard of care especially in early-stage tumors, this regimen may be complemented by adjuvant radio- or radio-chemotherapy in case of advanced and residual disease or the presence of certain histopathological risk factors [2–4]. The development of local tumor recurrences and regional or distant metastases has been shown to be the main prognostic factors in OSCC [3–6].

With surgical therapy being an essential part of the treatment algorithm as stated above, the achievement of free surgical margins is of highest relevance for the prevention of local disease recurrence and, thus, is strongly connected to patients’ survival. Subsequently, there have been numerous publications on the high correlation of the extent of resection margins with the incidence of disease recurrence and on optimal ranges of tumor resection with 3-5 mm being defined as safe minimum in most cases [7–12]. Intraoperative frozen section analysis is one tool to help optimizing the quality of resection and oncological outcome by preventing unnecessary resection and allow for immediate re-resection in cases of positive or close margins [13–15].

It is unclear whether quality of tumor resection in terms of clear margins is dependent of the affected subsite or not, in particular, when considering the complex three-dimensional anatomy and the limited access in the oral cavity. As there have been investigations on the high relevance of the site affected by the tumor for disease progression and survival showing distinct patterns of metastasis, disease recurrence and survival for each subsite of the oral cavity, the question arose if those differing courses of disease may be linked to the resection margins that had been achieved during primary therapy [16–19].

Therefore, the goal of the presented study was to evaluate the achieved resection margins in a large cohort of surgically treated patients with oral squamous cell carcinoma with regard to the affected subsites, the tumor stage, the results of the intraoperative frozen-section analysis and their impact on the corresponding recurrence and survival rates.

## Material and methods

### Study design

The presented study was designed as retrospective cohort study. All data were collected and analyzed in a single center (Department of Oral and Cranio-Maxillofacial Surgery, Heidelberg University Hospital, Heidelberg Germany). The study was approved by the Ethics Committee of the Medical Faculty of the University of Heidelberg (Ethic vote: S-183/2015) and conducted in full accordance with ethical principles and the Declaration of Helsinki. The presented manuscript was prepared according to the STROBE guidelines [20].

### Data collection

All patients with squamous cell carcinoma of the oral cavity (OSCC) and primary surgical treatment in the Department of Oral and Cranio-Maxillofacial Surgery of the Heidelberg University Hospital between the years 2010 and 2021 were included in this retrospective cohort study. Surgical therapy was planned and carried out after histological confirmation of OSCC and after completed tumor staging procedure including contrast agent CT of the head, neck and thorax. Patients with unspecific pulmonary findings without clear signs of malignancy received treatment with curative intent based on interdisciplinary tumor conference recommendations and were referred to short-term postoperative follow-up scans.

All included patients received resection of the primary tumor with adequate reconstruction and elective or therapeutic uni- or bilateral neck dissection in dependence of tumor stage, clinical neck status and affected subsite. Adjuvant radiotherapy or radio-chemotherapy was applied in cases of advanced tumors, incomplete resection, detection of lymph node metastases, or histopathological risk factors like perineural, lymphatic or vascular infiltration.

Clinical and pathological data were assessed retrospectively via the clinic’s patient management software (SAP, Walldorf, Germany). Clinical, pathological and demographic data were collected including the pathological tumor stage, the site of the primary tumor within the oral cavity, the results of the intraoperative frozen section analysis, the minimal resection margins in the main tumor specimen, the number of cases in which intraoperative re-resection was performed, and the achieved final resection status based on the main specimen and correlated re-resection specimens. The intraoperative frozen section analysis (IFSA) was being performed by the treating pathologist on the main tumor specimen. For the TNM data in this study, the 8th TNM classification system was used.

Survival data was collected and overall and progression-free survival were assessed with a focus on the aforementioned characteristics. Overall survival was defined as the period of time from initial therapy until the date of confirmed death or the last follow-up (censored data). Progression-free survival was defined as period of time from initial therapy to confirmed (pathologically or radiologically) local, regional or distant disease progression or last follow-up (censored data).

### Statistical analysis

Microsoft Excel 2013 (Microsoft, Redmond, WA, USA) and SPSS Statistics® 25 (IBM, Armonk, NY, USA) were used for the statistical analysis in the presented study. Receiver operating characteristic (ROC) curve analyses were used to estimate the prognostic impact of the resection margins in mm for progression-free survival in the different anatomical subsites. The Kaplan–Meier method was used for survival analysis and log-rank testing was applied to determine differences between the groups. Multivariate Cox regression analysis was used to analyse the impact of relevant clinical and pathological features on overall and progression-free survival. A *p*-value of 0.05 or less was considered to indicate statistical significance.

## Results

### Patient cohort

An overall number of 567 patients received surgical treatment of an oral squamous cell carcinoma in the Department of Oral and Maxillofacial Surgery in Heidelberg. Of those 567 patients, 125 had to be excluded from the analysis of exact resection margins because of missing data in the pathological reports due to a change in histopathological examination and documentation in our center during the early phase of the period that was included in our analysis. All patients, however, could be included in the comparison of the IFSA results with the final resection status.

235 (41.4%) were female and 332 (58.6%) were male. The mean age was 64.9 ± 12.1 years with a range from 19 to 95 years. All patients suffered from a squamous cell carcinoma of the oral cavity and received primary surgical treatment. 210 patients (37%) received additional adjuvant therapy via radio- or radio-chemo-therapy. The discrepancy between the number of patients suffering from advanced disease with an indication for adjuvant treatment (T3/4, N + ; *n* = 315 patients) and the number of patients actually receiving adjuvant treatment (*n* = 210 patients) may be explained by patient’s refusal, prolonged hospitalization due to postoperative complications, wound healing disorders etc.

Table 1 gives an overview of the demographical, clinical and pathological data of the patient cohort.

**Table 1:** Descriptive data regarding demographic and clinical features of the investigated cohort

Parameter	Number of cases (%)
Gender	
Female	235 (41.4%)
Male	332 (58.6%)
Age	
< 65 years	273 (48.1%)
> 65 years	294 (51.9%)
Pathological T Stage	
T1	191 (33.7%)
T2	148 (26.1%)
T3	47 (8.3%)
T4	181 (31.9%)
Pathological N Stage	
0	374 (66%)
1	56 (9.9%)
2a	4 (0.7%)
2b	57 (10.1%)
2c	33 (5.8%)
3	43 (7.5%)
M Stage	
0	564 (99.5%)
1	2 (0.5%)
Differentiation Grade	
1	52 (9.1%)
2	399 (70.4%)
3	94 (16.6%)
Missing	22 (3.9%)
Risk Factors	
Tobacco Consumption	
Yes	281 (49.6%)
No	286 (50.4%)
Alcohol Consumption	
Yes	203 (35.8%)
No	364 (64.2%)
Adjuvant Therapy	
Yes	210 (37%)
No	357 (63%)
Disease Recurrence	
Local recurrence	118 (20.8%)
Regional metastases	30 (5.3%)
Distant metastases	18 (3.2%)

### Main specimen resection margins as correlated to tumor subsite,stage, grading and histopathological infiltration parameters

Detailed data on the obtained resection margins of the main specimen were available for 442 patients. The resection margins were analyzed regarding the distribution of the primary tumors within the oral cavity. The different subsites affected by the tumors for the site-specific analysis included the tongue, the floor of the mouth, the maxilla, the mandibular alveolar process and the buccal mucosa.

**Fig. 1:**
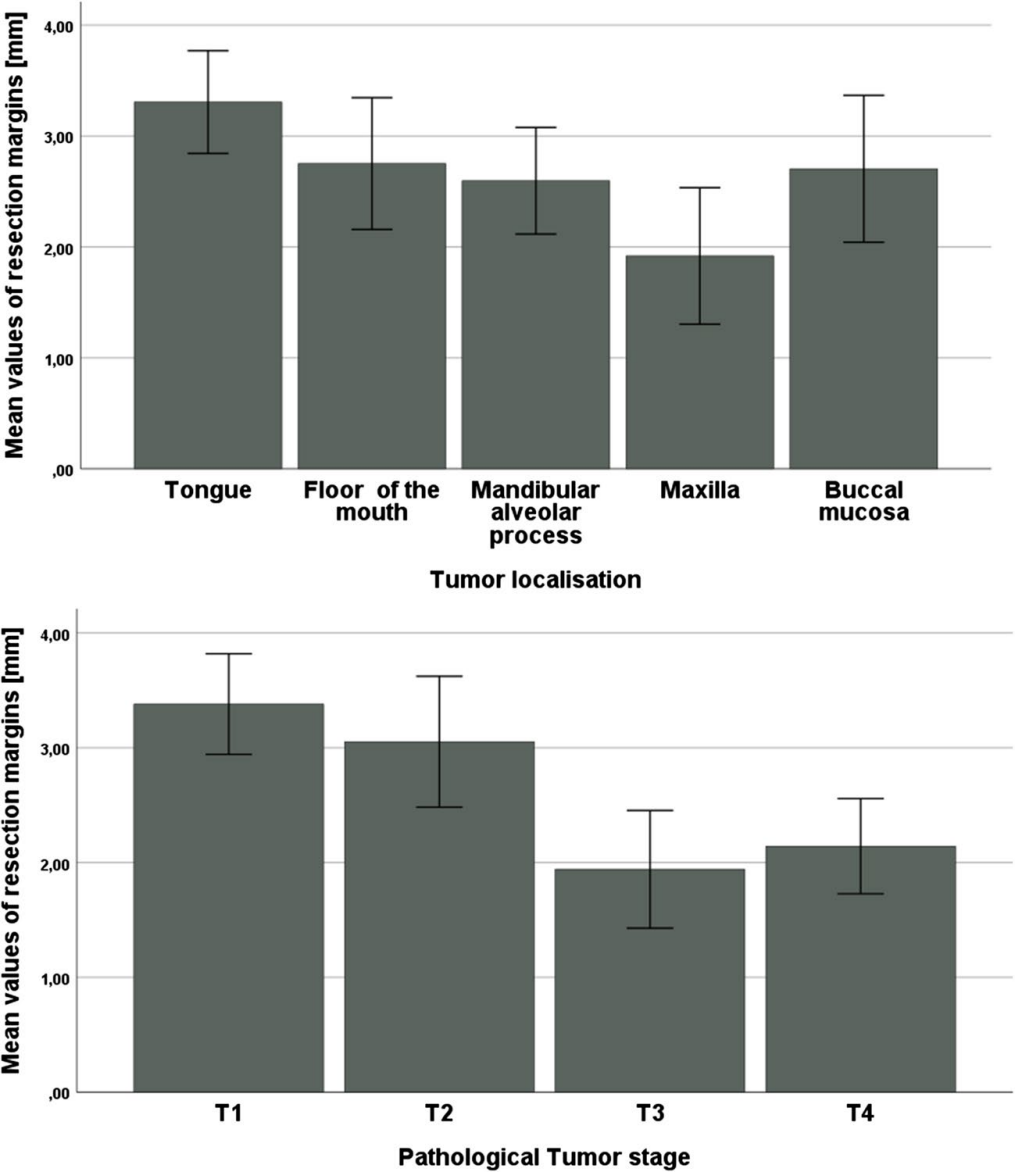
Bar charts depicting the mean values of resection margins in dependence of subsite of the oral cavity and Tumor Stage with 95%-CI

Here, the observed mean values of minimal resection margins differed significantly between the investigated subsites (*p* = 0.042) with the lowest values (i.e. closest resection margins) in the maxilla with 1.9 ± 2.3 mm and the highest values (i.e. widest resection margins) in the tongue with 3.3 ± 2.3 mm (Fig. 1, Table 2).

**Table 2:** Mean values of the resection margins or oral squamous cell carcinomas in dependence of the anatomical subsite of the oral cavity and of different T stages (for the whole cohort and subsite- and stage-specific analysis) for 442 patients with complete pathological reports (*p*-Value for one-way analysis of variance – ANOVA)

Subsite	*n*	Mean values (SD)	95-CI	Range (mm)	*p*-Value
Tongue	101	3.3 (± 2.3)	2.8–3.8	0–12	0.042
Floor of the mouth	111	2.8 (± 3.2)	2.2–3.3	0–26	
Mandibular alveolar process	130	2.6 (± 2.8)	2.1–3.1	0–13	
Maxilla	54	1.9 (± 2.3)	1.3–2.5	0–10	
Buccal mucosa	46	2.7 (± 2.2)	2.0–3.4	0–8	
T stage	*n*	Mean values (SD)	95-CI	Range (mm)	*p*-Value
T1	131	3.4 (± 2.5)	2.9–3.8	0–12	< 0.001
T2	115	3.1 (± 3.1)	2.5–3.6	0–26	
T3	44	1.9 (± 1.7)	1.4–2.5	0–7	
T4	152	2.1 (± 2.6)	1.7–2.6	0–13	
All subsites and Stages	442	2.7 (± 2.7)	2.5–3.0	0–26	
Distinct analysis of resection margins as related to subsite and stage					
	*n*	Mean values (SD)	95-CI	Range (mm)	*p*-Value
Tongue					
T1	45	3.8 (2.5)	3.0–4.5	0.1–12	0.09
T2	32	3.3 (2.3)	2.5–4.1	0–10	
T3	20	2.2 (1.7)	1.4–3.0	0.1–5	
T4	4	3.3 (3.3)	-1.9–8.5	0.1–7	
Floor of the mouth					
T1	40	3.1 (2.7)	2.2–4.0	0–11	0.24
T2	41	3.1 (4.2)	1.8–4.5	0–26	
T3	7	1.5 (1.5)	0.1–2.9	0.1–4	
T4	23	1.8 (1.7)	1.1–2.5	0–5	
Mandibular alveolar process					
T1	19	2.6 (2.4)	1.4–3.7	0–6	0.79
T2	18	3.1 (2.6)	1.8–4.4	0–8	
T3	5	3.2 (2.3)	0.4–6.0	1–7	
T4	88	2.5 (2.9)	1.8–3.1	0–13	
Maxilla					
T1	10	3.7 (2.8)	1.7–5.7	0–10	0.01
T2	7	2.6 (2.3)	0.5–4.7	0–6	
T3	7	0.6 (0.7)	0–1.3	0–2	
T4	30	1.5 (2.0)	0.7–2.2	0–6	
Buccal mucosa					
T1	18	3.6 (2.2)	2.5–4.7	0–8	0.09
T2	17	2.5 (2.1)	1.4–3.6	0.1–6	
T3	5	2.0 (1.5)	0.1–3.9	0.5–4	
T4	6	1.4 (2.2)	-0.6–3.4	0–5	

Furthermore, the resection margins were analyzed regarding their distribution in dependence of the different T stages of the primary tumors. The mean resection margins differed significantly (T1: 3.4 mm; T2: 3.1 mm; T3: 1.9 mm; T4: 2.1 mm) (Fig. 1 and Table 2).

Additionaly, we conducted an analysis of the mean resection margins in dependence of tumor grading and the existence of perineural, lymphatic and vascular infiltration in the pathological reports. While we did find differences in mean resection margins in dependence of grading, those differences were not statistically significant (*p* = 0.1). Resection margins of tumors with signs of perineural invasion differed significantly (*p* = 0.002), while those with signs of lymphatic or vascular infiltration did not show significant differences. Table 3 depicts the data of the analysis of resection margins in dependence of tumor grading and histopathological infiltration parameters.

**Table 3:** Correlations of findings in the intraoperative frozen section analysis and of the final resection status with T stage, and affected subsite

Tumor Characteristics	*n*	Mean values (SD)	95-CI	Range (mm)	*p*-Value
Grading					
G1	36	3.6 (2.6)	2.7–4.5	0–10	0.1
G2	322	2.6 (2.7)	2.3–2.9	0–26	
G3	75	2.8 (2.7)	2.1–3.4	0–13	
Lymphatic infiltration					
L0	203	2.7 (2.3)	2.4–3.0	0–11	0.1
L1	107	2.2 (2.3)	1.8–2.7	0–10	
Vascular infiltration					
V0	265	2.5 (2.2)	2.2–2.7	0–11	0.2
V1	18	1.8 (2.3)	0.7–3.0	0–6	
Perineural infiltration					
Pn0	147	2.9 (2.4)	2.5–3.2	0–11	0.002
Pn1	79	1.9 (1.9)	1.4–2.3	0–7	

### Intraoperative frozen section analysis (IFSA)

We investigated the rates of close margin resections or incomplete tumor resections in the intraoperative frozen section analysis in our cohort. Pathological reports with information on the results of the IFSA and final resection status without exact margins in mm were available for 545 patients. Free margins were reported for 458 patients (80.8%) by IFSA, close margin or incomplete resection was reported by IFSA in 87 patients (15.3%), the rest of the pathological reports did not contain specific information on IFSA results that were reported intraoperatively. We found a sensitivity of 66% and a specificity of 100% for IFSA in our analysis. Of the 458 patients with clear resection in IFSA, 35 patients (7.6%) were then classified as incomplete or close margin resection in the final pathological report. There were no patients with positive or close margins in IFSA whose IFSA specimen were declared negative afterwards.

Furthermore, we found significant correlations of the IFSA results with pathological T stage, final resection status and anatomical tumor localization (Table 4).

**Table 4:** Correlations of findings in the intraoperative frozen section analysis and of the final resection status with T stage, and affected subsite

Intraoperative frozen section analysis			
	Clear margins (IFSA)	Close/involved margins (IFSA)	*p*-Value
T stage			
T1	174 (94.6%)	10 (5.4%)	< 0.001
T2	125 (88%)	17 (12%)	
T3	35 (74.5%)	12 (25.5%)	
T4	124 (72.1%)	48 (27.9%)	
Subsite			
Tongue	117 (90.7%)	12 (9.3%)	0.04
Floor of the mouth	119 (84.4%)	22 (15.6%)	
Mandibular alveolar process	137 (82.5%)	29 (17.5%)	
Maxilla	43 (72.9%)	16 (27.1%)	
Buccal mucosa	42 (84%)	8 (16%)	
Final resection status			
R0	423 (87.4%)	61 (12.6%)	< 0.001
R + (R1, R2, Rx)	35 (57.4%)	26 (42.6%)	
Final resection status			
	Clear margins	Close/involved margins	*p*-Value
T stage			
T1	184 (96.3%)	7 (3.7%)	< 0.001
T2	138 (93.2%)	10 (6.8%)	
T3	43 (91.5%)	4 (8.5%)	
T4	134 (74%)	47 (26%)	
Subsite			
Tongue	132 (99.2%)	1 (0.8%)	< 0.001
Floor of the mouth	135 (92.5%)	11 (7.5%)	
Mandibular alveolar process	142 (81.6%)	32 (18.4%)	
Maxilla	44 (71%)	18 (29%)	
Buccal mucosa	46 (88.5%)	6 (11.5%)	

### Resection status

Resection status of the main specimen of all patients was analyzed and correlated to tumor stage and subsite. Table 4 presents the results of the correlation analysis of final resection status with T stage and oral cavity subsite. While the exact margins in mm refer to the main resection specimen, the final resection status considers the re-resection specimens as correlated by the responsible pathologist and surgeon.

Intraoperative re-resection was performed in 131 patients (23.1%). In 38 of these patients (29%), no tumor could be detected in re-resection specimens, although IFSA reported on involved margins in the first resection specimen.

### Disease recurrence and survival rates

**Fig. 2:**
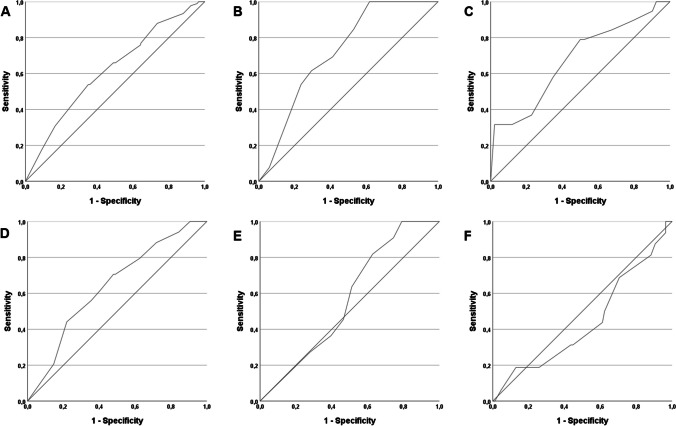
ROC graphs illustrating the prognostic relevance of the resection margins in mm on local recurrence-free survival for patients with oral squamous cell carcinoma in different anatomical subsites: (A) All subsites, AUC = 0.625 (95%-CI: 0.561–0.689), *p* < 0.001; (B) Buccal mucosa, AUC = 0.722 (95%-CI: 0.575–0.868), *p* = 0.02; (C) Floor of the mouth, AUC = 0.675 (95%-CI: 0.537–0.813), *p* = 0.017; (D) Mandibular alveolar process, AUC = 0.641 (95%-CI: 0.537–0.745), *p* = 0.015; (E) maxilla, AUC = 0.568 (95%-CI: 0.401–0.734), *p* = 0.49; (F) Tongue, AUC = 0.440 (95%-CI: 0.283–0.598), *p* = 0.451 (ROC, receiver operating characteristic; AUC, area under the ROC curve)

ROC analyses were performed for the whole cohort and for each distinct subsite of the oral cavity to determine the predictive value of the extent of resection (margins in mm) for the development of local tumor recurrences. Here, we could confirm the significant correlation of the extent of surgical margins with local control rates for the whole cohort and for most of the subsites of the oral cavity, except for the tongue and the maxilla, although AUC and *p*-Values for the different subsites differ (Fig. 2). Our analysis showed significant correlations between affected subsites and rates of local disease recurrence (Table 5; *p* = 0.04) and the rates of adjuvant radiotherapy (*p* < 0.001).

**Table 5:** Local recurrence rates in dependence of subsite of the oral cavity affected by the primary tumor (*p*-Value according to chi-squared testing)

Subsite	No local recurrence	Local recurrence	*p*-Value
Tongue	115 (86.5%)	18 (13.5%)	0.04
Floor of the mouth	124 (84.9%)	22 (15.1%)	
Mandibular alveolar process	113 (74.7%)	44 (25.3%)	
Maxilla	51 (82.3%)	11 (17.7%)	
Buccal mucosa	39 (75%)	13 (25%)	

**Fig. 3:**
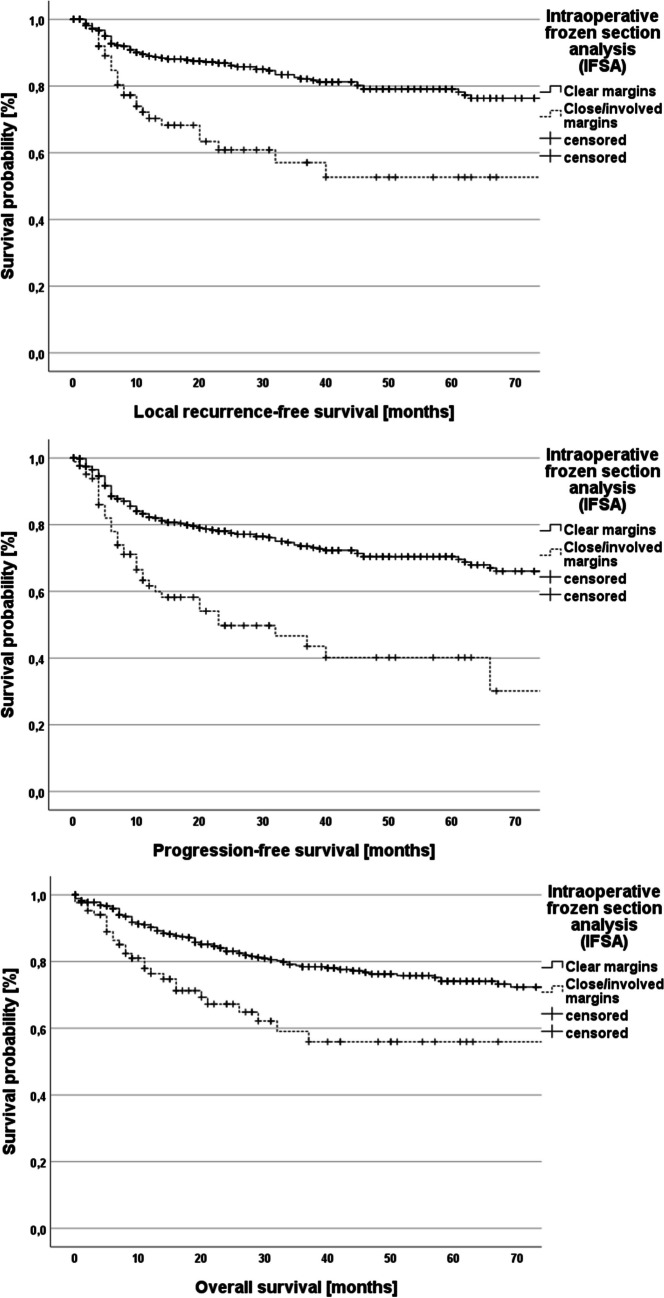
Kaplan–Meier plots demonstrating local recurrence-free survival, progression-free survival and overall survival in dependence of the results of the intraoperative frozen section analysis (log-rank test for all three plots: *p* < 0.001)

**Fig. 4:**
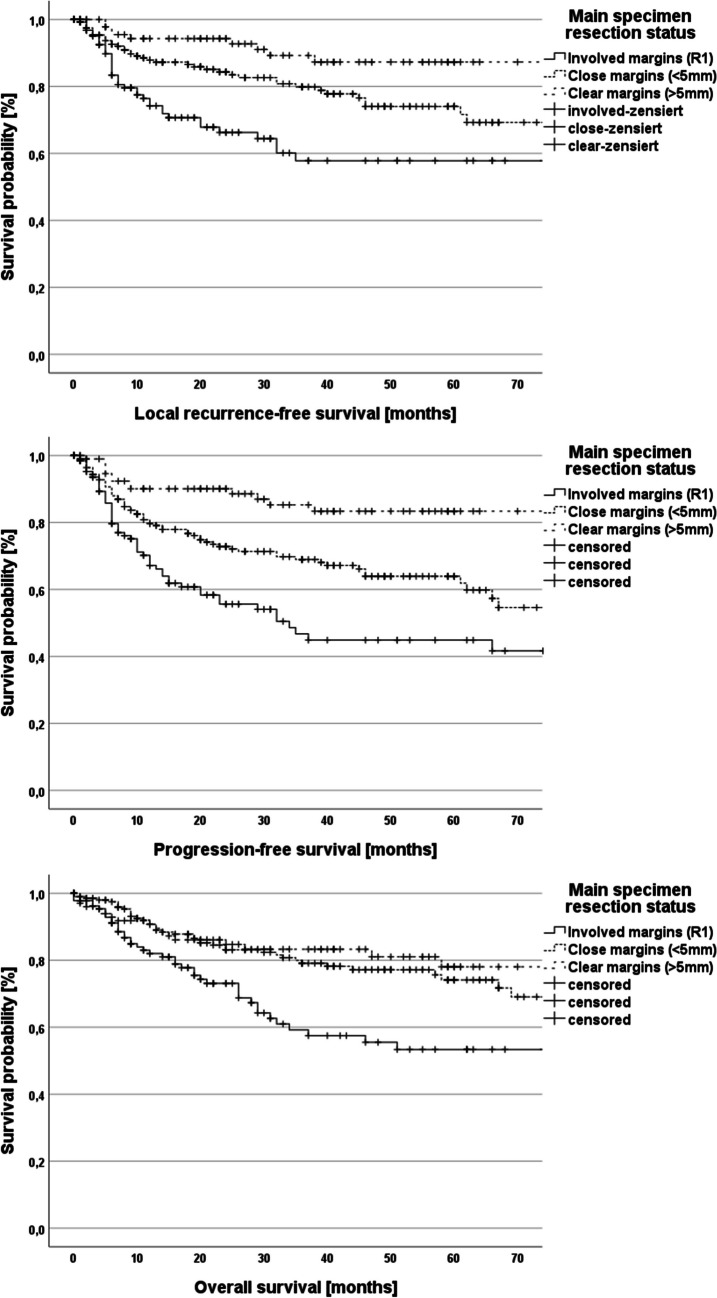
Kaplan–Meier plots demonstrating local recurrence-free survival, progression-free survival and overall survival in dependence of the main tumor specimen resection status (involved margins – R1, close margins < 5 mm, clear margins – R0; > 5 mm). Local recurrence-free survival: log-rank test: *p* < 0.001; Progression-free survival: log-rank test: *p* < 0.001; Overall survival: log-rank test: *p* = 0.002

**Fig. 5:**
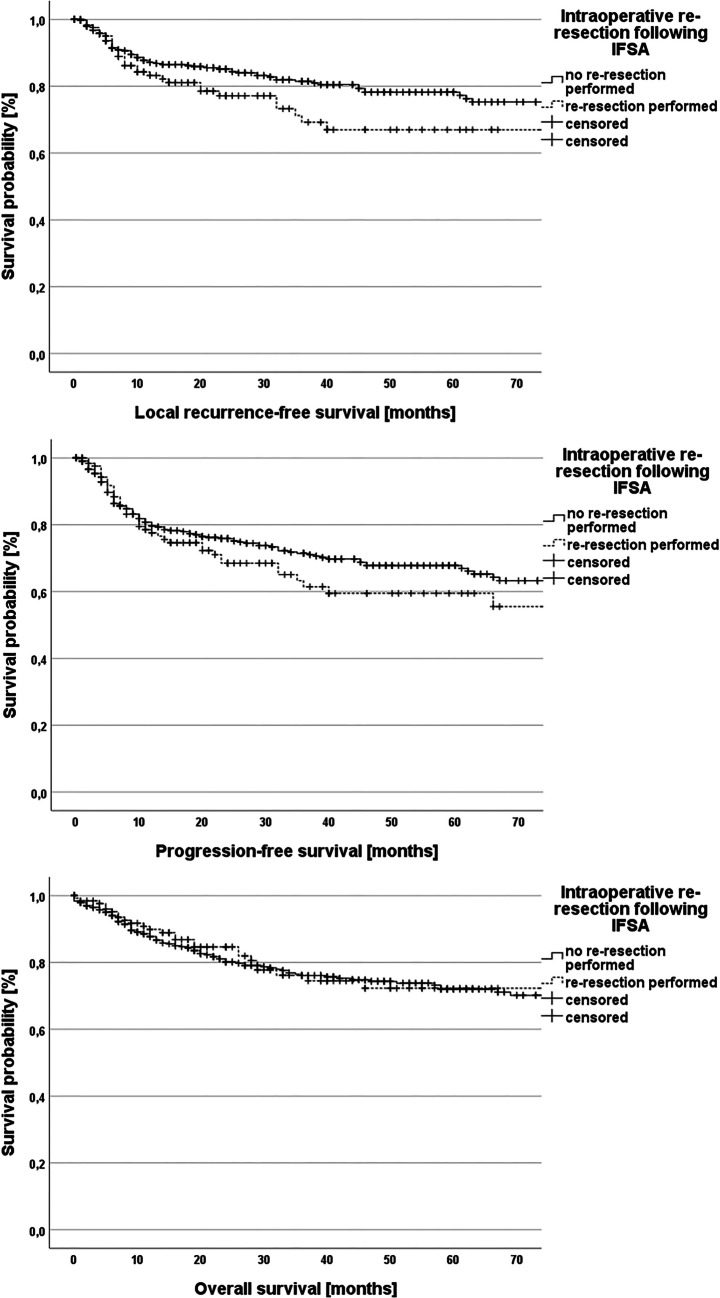
Kaplan–Meier plots demonstrating local recurrence-free survival, progression-free survival and overall survival in dependence of the performance of intraoperative re-resection. Local recurrence-free survival: log-rank test: *p* = 0.06; Progression-free survival: log-rank test: *p* = 0.27; Overall survival: log-rank test: *p* = 0.65

Univariate survival analysis was performed in dependence of IFSA results and final resection status and a significant impact of both variables on progression-free and overall survival could be confirmed (*p* < 0.001; Figs. 3 and 4). Moreover, the prognostic impact of the execution of intraoperative re-resection was analyzed (local recurrence-free survival – *p* = 0.06, progression-free survival – *p* = 0.27, overall survival – *p* = 0.65; Fig. 5).

## Discussion

Complete tumor resection with the achievement of free margins is one of the most relevant physician-dependent factors regarding long-term treatment success in oncological surgery. Several authors advocated resection margins between 3 and 5 mm as safe minimum in the surgical treatment of oral squamous cell carcinoma, while only a minority of publications did not show a relevant of connection of resection margins with survival [7–10, 21]. In the presented study, we investigated the surgical margins of the main resection specimen including intraoperative frozen section analyses (IFSA) in a large cohort of patients with squamous cell carcinomas of the oral cavity. The achieved margins were analyzed regarding their association with the affected subsite within the oral cavity, tumor stage and their impact on patients’ survival. As the clinical routine in our center includes intraoperative pathological examination of resection in terms of IFSA on the main resection specimen, we decided to focus on the quality of the initial tumor resection without considering intraoperative re-resections in dependence of IFSA results.

Not surprisingly, the delineated correlation of the extent of resection with the development of local tumor recurrence and, subsequently, with patients’ survival could be confirmed in our study and supports the validity of our data. In contrast to most of the mentioned studies, however, besides supporting the necessity of complete tumor resection, our study aimed on investigating subsite-specific differences in resection quality and their consequences for further treatment and patients’ outcome. Here, our analysis revealed significant differences in resection ranges between different subsites of the oral cavity and tumor size with highest mean values of resection margins in T1 tumors and tumors of the tongue, and lowest mean values for T3 tumors and maxillary tumors. These findings are of high clinical relevance, as they suggest different surgical approaches in dependence of T stage and affected subsite. SCC of the maxilla for example seem to bare a greater risk of insufficient tumor resection compared to the tongue. This observation is in line with the higher rates of local recurrences in subsites with a tendency towards closer resection margins in our analysis. However, the anatomical conditions in maxillary SCC (affection of the maxillary sinus, transition to retromaxillary space etc.) often aggravate the definition of clear resection for the pathologists, and, thus interpretability of the reported resection rates varies between the different subsites and tumor stages.

A weakness of studies on resection margins is the difficulty of data interpretation reviewing different pathological reports in cases of intraoperative frozen section analysis and consecutive re-resections. Nentwig et al. reported on the tendency towards closer final margins if IFSA revealed positive margins and the subsequently worsening of disease-free and overall survival [13]. We could confirm this finding with our data from a large cohort of patients. Here, the data imply relevant rates of insufficient re-resections after IFSA, and, subsequently illustrate the relevance of achieving clear margins during the initial resection. A reason for this phenomenon may be a loss of information on the exact position of remaining tumor tissue or the area of close resection between the treating surgeon and the examining pathologist. This problem probably is of higher relevance in larger tumors with complex three-dimensional extent which subsequently implies aggravated orientation. Aptly, our analysis showed a clear tendency towards a higher prognostic significance of the IFSA results in advanced tumors (T3/4, data not shown).

The intraoperative assessment of tumor boundaries and the achievement of clear resection margins is a known problem in oncological surgery and several techniques have been described to address this issue. Besides intraoperative frozen section analysis which may be seen as standard-of-care in most centres, navigation-assisted tumor resection and the use of autofluorescence-guided resection are two further tools that, among others, have been discussed and promoted lately [22, 23]. While those techniques may possibly have a beneficial impact on resection quality, they up to date are still limited to certain operative situations. So, for instance, navigation-based resection requires surrounding osseous structures as references to allow for reliable navigation and the use of autofluorescence is limited to the mucosa of accessible and flat areas of the oral cavity [22].

While the tendency towards higher rates of incomplete or close resections in advanced tumors may be possibly explained by limitations of resection due to functional or anatomical reasons (e.g., to avoid functional glossectomy, proximity to internal carotid artery etc.), we also found significantly differing resection margins in dependence of the oral cavity subsite affected by the primary tumor. A reasonable explanation for this observation could be a difference in tissue shrinkage after resection and in the course of pathological processing. This theory has been discussed by other authors and may be responsible for a part of the observed discrepancies [24–27]. It seems comprehensible that contractile soft tissue from the tongue or the buccal mucosa displays a different pattern of shrinkage than tissue from the hard palate. Also, Cheng et al. reported on higher rates of shrinkage in advanced tumors (T3/T4), which seems in line with our results, considering the lower minimal margins in T3/T4 tumors in our analysis [25]. As a consequence, it could be argued that the general recommendation of 1 cm resection range around the visible tumor may be insufficient and that the width of resection should consider the tumor stage and the affected subsite. Moreover, there are other means of determining the aggressiveness of a tumor that should be evaluated and considered in resection planning. For example, we did see a tendency towards closer resection margins in G2 and G3 tumors in comparison to G1 tumors, although the differences were not statistically significant. When it comes to histopathological infiltration parameters, we did find significantly smaller resection margins in tumors with signs of perineural infiltration, while tumors with lymphatic or vascular infiltration did not show significant differences in resection width in our analysis.

The reported retrospective data may form the base of prospective trials to evaluate this hypothesis in order to further enhance the outcome for affected patients. On the other hand, the relevant differences between resection margins and local recurrence rates observed in the tongue and in the buccal mucosa suggest discrepancies in accessibility for tumor resection or differing tumor growth patterns. While there have been publications advocating different levels of aggressiveness of tumors depending on the distinct subsite of presentation within the oral cavity, to the best of our knowledge, those reports have not been supported by robust clinical and/or biological data [28–30]. Moreover, despite the reported differences in minimal resection margins and rates of local recurrences, our data did not show a statistically significant difference in overall survival in dependence of the anatomical subsite affected by the tumor. This fact, however, may possibly be explained by the success of adjuvant therapy that was administered in cases of incomplete or close tumor resection, which is supported by the relevantly differing rates of adjuvant therapy in dependence of the oral cavity subsites affected by the tumors.

## Conclusions

The resection status is a highly relevant parameter for the oncological outcome of patients suffering from oral cancer. The data revealed that the quality of resection may vary depending on the particular subsite of the oral cavity,the tumor size and tumor growth patterns, determined by grading and histopathological infiltration parameters. Furthermore, the results of the intraoperative frozen section analysis significantly correlate with patients’ survival, indicating inadequate intraoperative re-resections, especially in advanced tumors. This further emphasizes the urgent need for additional tools to help the surgeon in achieving optimal resection results. While intraoperative frozen section analysis has become a standard in most centres, further techniques to improve the quality of resection are warranted.
